# The Maternal Vaccine Study Protocol: A Victorian Cohort Study Evaluating Infant and Childhood Safety and Health and Developmental Outcomes After Vaccination Against Respiratory Viruses in Pregnancy

**DOI:** 10.3390/vaccines14050449

**Published:** 2026-05-18

**Authors:** Rachael Purcell, Margie Danchin, Nigel W. Crawford, Eric Zhao, Ashleigh Rak, Michelle L. Giles, Jim Buttery

**Affiliations:** 1 Epidemiology Informatics, Murdoch Children’s Research Institute, Melbourne, VIC 3052, Australia; 2 Department of Paediatrics, The University of Melbourne, Melbourne, VIC 3010, Australia; 3 Centre for Health Analytics, The Royal Children’s Hospital, Parkville, VIC 3052, Australia; 4 Vaccine Trials and Uptake Group, Murdoch Children’s Research Institute, Melbourne, VIC 3052, Australia; 5 Department of General Medicine, Royal Children’s Hospital, Melbourne, VIC 3052, Australia; 6 SAEFVIC, Murdoch Children’s Research Institute, Melbourne, VIC 3052, Australia; 7 Department of Obstetrics and Gynaecology, Monash University, Melbourne, VIC 3168, Australia; 8 Department of Infectious Diseases, The University of Melbourne, Melbourne, VIC 3052, Australia

**Keywords:** maternal vaccination, pregnancy, vaccine, infant health, development, allergy

## Abstract

Objectives: Changes in public policy are eroding vaccine confidence. Previously accepted peer-reviewed evidence around vaccination and developmental outcomes for children is being questioned. Robust, methodologically sound safety data are more needed than ever to maintain consumer confidence. Establishing further safety data on infant health, development, and allergies after COVID-19 and influenza vaccination in pregnancy may improve confidence and acceptance. Methods: This is a state-wide multi-centre prospective cohort study conducted as a sub-study of the Generation Victoria birth cohort. It will examine the risk difference for infant health, developmental, and allergy outcomes between groups of mother–baby pairs who will be examined according to exposure (vaccination against a respiratory virus during pregnancy) and comparator (no vaccination against a respiratory virus). Results: Data contributing to the analysis include GenV-collected developmental, health, and allergy outcomes to 12 months of age, as well as data from state-wide linked datasets. Conclusions: This linked-data longitudinal study will provide information on health, allergy, and developmental outcomes for infants in the first year of life after influenza and COVID-19 vaccination during pregnancy. Implications for Public Health: The reporting of developmental data will be a new contribution to knowledge around outcomes after vaccination during pregnancy.

## 1. Introduction

Changes in public policy internationally risk eroding confidence in vaccine safety globally and in Australia [1]. Previously accepted peer-reviewed evidence regarding vaccination and subsequent developmental outcomes for children is being challenged [2]. Accompanied by the “infodemic” proliferation of vaccine misinformation on social media that accelerated during the COVID-19 pandemic, threats to existing and new vaccine programs represent a clear danger to a public health intervention that has averted more than 154 million deaths since 1974 [3]. There is an increasing need for robust, methodologically sound safety data to sustain consumer confidence. Describing outcomes that are key areas of concern for families, such as child development and allergy, is needed.

Compounded by changes in vaccine policy is the risk of severe disease that priority populations face, particularly against vaccine-preventable respiratory viral infections. This risk is further heightened during pandemics. This has been demonstrated by the higher mortality and critical illness rates in unvaccinated pregnant women during the COVID-19 pandemic [4]. The complications of COVID-19 disease in unvaccinated pregnant women are particularly prominent in the second half of pregnancy [5,6]. Pregnant women infected with SARS-CoV-2 are also at higher risk of adverse fetal outcomes, including preterm birth [7].

Safe and efficacious vaccine development has been a cornerstone in the control and management approach to the COVID-19 pandemic. COVID-19 vaccine uptake in Australia has been a public health success story, with 94% of adults and young people aged 12 years and over vaccinated with a two-dose primary series. However, pregnant persons, who were at high risk of severe COVID-19 disease at the beginning of the pandemic, had lower vaccination coverage when compared with the general Australian population [8,9]. Contributors to this were the exclusion of pregnant women from vaccine trials, delays in recognising pregnant persons as a priority population, and concerns around safety data [10].

There are a number of vaccinations recommended during pregnancy to protect mother and infant, including influenza, pertussis, COVID-19, and, most recently, respiratory syncytial virus (RSV) [11]. Whilst there are studies describing pregnancy and health outcomes after influenza vaccination during pregnancy [12,13], more data are needed on health outcomes after COVID-19 maternal vaccination. Developmental outcomes for infants and children have not been described for COVID-19 or influenza vaccines administered in pregnancy. There are also high levels of parental concern around the development of childhood allergy [14], and data demonstrating the safety of maternal vaccination may be of community value. In an environment where vaccine recommendations in other jurisdictions are politically motivated and vaccine confidence is being eroded, safety data and outcomes for children after maternal vaccination during pregnancy are critical.

This is the study protocol for the Maternal Vaccine study. The primary objective is to investigate child health, developmental, and allergy outcomes at 12 months of age after maternal vaccination with COVID-19 and/or influenza vaccines during pregnancy. This will be addressed through a state-wide longitudinal cohort study, Generation Victoria (GenV), based in Victoria, Australia [15,16], and a linked-data analysis. The GenV study recruited over 40,000 mother–baby pairs from 04/10/2021 to 03/10/2023 and collected parental input data at time points across infancy. These data will be linked to statewide datasets, including the Victorian Admitted Episodes Dataset (VAED), the Victorian Emergency Minimum Dataset (VEMD), and the national Australian Immunisation Register (AIR). Secondary objectives will include a sub-analysis examining factors such as the trimester of vaccination, the type of COVID-19 vaccine received, maternal demographic characteristics, and co-administration with other recommended maternal vaccines such as pertussis.

## 2. Methods

### 2.1. Eligibility Criteria, Study Design, and Study Period

The study population will include women and infants who have consented to be enrolled in the GenV study based in Victoria [16]. Study data will be analysed using a linked dataset comprising GenV data, AIR [17], VEMD, and VAED [18]. Only mother–baby pairs who can be linked to the Australian Immunisation Register (AIR) will be included for analysis. Families who subsequently withdraw their GenV study consent and mother–baby pairs who are unable to be data-linked will be excluded. Neonatal and infant data relevant to the primary and secondary outcomes of our study were collected from the time of enrolment through to 12 months of age, ending 3 October 2024 [16].

Data to be analysed will include the following:

1. Maternal receipt of a COVID-19, influenza, or pertussis vaccination status from AIR [17], including gestational age at maternal vaccination and number of vaccinations received;
2. Infant gestational age at the time of birth using ICD-10-AM [19] administrative coding from VAED [18];
3. Maternal and infant demographics (Table 1);
4. Developmental outcomes collected at 3, 6, 9, and 12 months of age via the GenV and Me platform [16];
5. Health outcomes collected at 3, 6, 9, and 12 months of age via the GenV and Me platform [16];
6. Infant allergy outcomes collected at 12 months of age via the GenV and Me platform [16];
7. Health and allergy outcomes for infants up to the age of 12 months as defined by administrative coding endpoints (ICD-10-AM) [19] within the Victorian Admitted Episodes Dataset [18] and the Victorian Minimum Emergency Dataset [20].

Exposure: The exposure(s) of interest are COVID-19 and influenza vaccines received by a mother during the current pregnancy. The exposures will comprise the groups detailed in Table 2. Vaccine exposure will be determined by linkage of the maternal immunisation record (AIR) to the mother–baby pair. All vaccines of interest were available and recommended during pregnancy in Victoria during the study period.

Comparator: The comparator, or unexposed groups, will include mother–baby pairs whereby the mother (1) did not receive any vaccines during pregnancy, or (2) received a non-respiratory virus vaccine (pertussis) during pregnancy. The rationale for two comparator groups is to minimise biases that may be present in a completely non-vaccinated cohort, and the maternal pertussis vaccine is the highest maternal vaccine coverage historically in Victoria [21,22]. The pertussis-only vaccinated group will be used as the main reference category, and the completed unvaccinated group will be used in secondary analyses as an alternative comparator.

Outcome: The study will estimate the difference in proportion (i.e., risk difference) of pre-specific infant health and developmental outcomes between women exposed to a COVID-19 and/or influenza vaccine and non-exposed women during pregnancy for 12 months post-delivery.

Core perinatal and infant health outcomes will include: preterm birth, defined by birth prior to 37 weeks of gestation; major congenital malformation, defined as a malformation identified by parents and/or health practitioners within the capabilities of the GenV dataset; small for gestational age infant defined as an infant born at <10th centile for gestational age; neonatal hospitalisation within the first 28 days of life; and neonatal death within the first 28 days of life.

Core childhood health outcomes to be assessed include the following: allergies, defined as a drug or food allergy identified by a parent or healthcare practitioner within the GenV dataset; medical conditions, defined as any medical condition identified by parents or healthcare providers within the GenV dataset; hospitalisation during the 12-month study period (e.g., from the date of birth, until 365 days later). The Victorian Admitted Episodes Dataset (VAED) and Victorian Emergency Minimum Dataset (VEMD) [18] will be searched for ICD-10-AM [19] codes reflecting hospitalisation in a Victorian Health service relating to allergy, infection, and immunity (Table 3).

Core childhood developmental outcomes will be collected via parent-administered “GenV and Me” questionnaire data at pre-specified time points as a part of the GenV study [16]. These include core phenotypic outcomes such as senses (hearing and vision), fine and gross motor, learning, language, cognition, and mental health and behavioural milestones [23], assessed at 3, 6, 9, and 12 months using a digital platform [16]. Outcomes are collected using a five-point scale asking for parental perception of their child’s development. A limitation of this methodology is that developmental outcomes based on parent-reported data may be subject to measurement error and reporting bias. Misclassification of outcomes is possible, including non-differential misclassification, which may bias estimates towards the null, and differential misclassification if reporting differs by vaccination status. These limitations will be considered in the interpretation of the findings. To minimize loss to follow-up, guardians are sent reminder emails by the GenV study at each data collection time point. In the event of incomplete survey completion by parents, a responder bias may also be possible.

Socioeconomic status will be assigned using the postcode available for the family residence, using the Index of Relative Socio-Economic Advantage and Disadvantage (SEIFA) score, derived by the Australian Bureau of Statistics [24]. SEIFA scores will be categorised into those in the upper 80% of scores and those in the lower 20% of scores [25].

### 2.2. Identification of the Pregnancy Exposure Period

**Figure 1:**
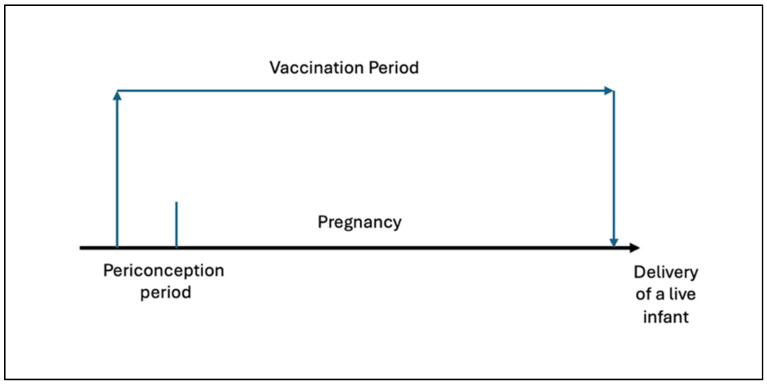
Vaccination exposure during the pregnancy period.

Receipt of a relevant vaccine during pregnancy will be determined through data linkage between the maternal date of vaccination recorded on the Australian Immunisation Register and the gestational age of the infant recorded through either the GenV and Me survey, or ICD-10-AM [19] coding in the VAED [18], or an end-of-pregnancy event code recorded in the maternal VAED [18] record. For the purpose of determining if an exposure event (vaccination) occurred during pregnancy, the pregnancy exposure period will be defined by subtracting the gestational age (presumed to be 40 weeks unless otherwise specified) minus 14 days to 1 day prior to birth (Figure 1) [26].

### 2.3. Gestational Age, Preterm Birth, and Developmental Outcomes

Gestational age at the time of birth will be determined by the ICD-10-AM [19] birth codes recorded in the VAED for each participant. If the gestational age is less than 37 weeks, gestational age will be grouped into gestational ranges consistent with ICD-10-AM Version 11 (Supplementary Materials) [19]. Preterm birth will be corrected for when analysing developmental outcomes for participants with a gestational age of less than 37 weeks. A sensitivity analysis of parental reporting of gestational age at the 9-month GenV and Me survey will be conducted.

### 2.4. Statistical Analysis Plan

Categorical variables will be summarised using numbers and percentages. Continuous variables will be summarised using mean and standard deviation, or median and interquartile ranges, depending on the distribution. To address the primary objective, binomial regressions (generalised linear model) will be used to estimate the difference in proportions (risk difference) of outcomes between each of the exposed groups and the non-exposed group (as the reference). These models assume a binomial distribution of the outcome and independence of observations between individuals. Risk difference is chosen because it applies an absolute measure of effect that is directly interpretable as the difference in outcome risk between exposure groups [27]. If there are imbalances between the exposed and unexposed groups of more than 20 percent, an additional “unexposed” group comprising women who have only received pertussis vaccination during pregnancy may be considered. A standardised difference of >0.2 (20%) is chosen to identify a moderate imbalance that may meaningfully affect comparability between groups [28].

**Figure 2:**
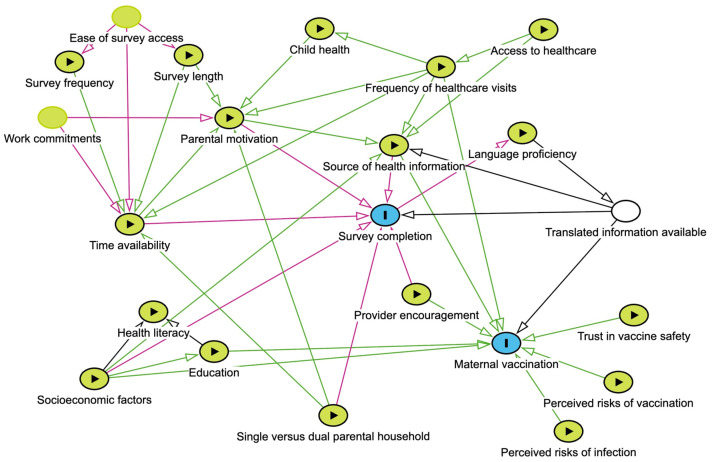
Causal diagram considering factors influencing guardian GenV and Me survey completion and maternal vaccination. Blue circles represent outcomes. Green circles represent exposures which may influence the outcome. White circles represent adjusted variable.

To address the secondary objective, a detailed directed acyclic graph has been developed, identifying the types of relationships the variables have with the outcome and exposure (Figure 2). In line with the directed acyclic graph, the variables will be either used as a covariate in the model (for adjusting confounding effect) or analysed separately (for interaction effect), or others as necessary. For repeated measures, repeated measures generalised linear models will be used. Covariates will be selected a priori based on subject-matter knowledge and informed by the directed acyclic graph (Figure 2) with the aim of identifying a minimally sufficient adjustment set to control for confounding.

For the analysis of developmental outcomes, two strategies will be used. A primary analysis will analyse participants who have data at multiple time points recorded by guardians. Developmental trajectory will be assessed, and the risk difference will be described between the exposure groups. A mixed-effects linear regression model will investigate associations between the exposures (vaccination) and outcome (development) [29]. A mixed-effect linear regression model was chosen to account for the longitudinal structure of the data. This allows for the inclusion of fixed effects (e.g., vaccination) and random effects (e.g., individual-level variability) to account for within-child correlation over time.

Model assumptions and fit will be assessed for all regression models. This will include examination of residuals and comparison of model fit using criteria such as AIC. For mixed-effects models, residual diagnostics and the distribution of random effects will also be assessed.

For participants who have developmental outcomes recorded by multiple guardians, we will conduct a sensitivity analysis to determine if these results differ from those with input from a single guardian. For participants whose guardians complete the GenV and Me assessment at a single time point, a separate analysis examining risk difference will be completed.

For outcomes measured at multiple time points, regression models with cluster-robust standard errors of mixed-effects models will account for correlation within individuals. Family-level clustering, for example, siblings, and geographical clustering will be accounted for using cluster robust standard errors of multilevel models. All analyses will be conducted in R [30].

Study findings will be presented using a combination of graphical images and tables. Summaries of baseline characteristics and outcome estimates will be presented in tables. Graphical methods, including forest plots for effect estimates and line plots for longitudinal outcomes, will be used to aid interpretation and visualisation of results.

### 2.5. Management of Missing Data

Due to the large number of participants and longitudinal nature of this study, it is anticipated that there may be a number of participants with missing data across different data collection points. Several factors may influence the likelihood of parental survey completion (the outcome of interest) (Figure 2).

Depending on the volume and pattern of data, missing data may not occur at random (MNAR). In this scenario, inverse-probability weighting could be applied with an MNAR sensitivity analysis conducted. Table 4 further outlines an approach to missing data based on the outcomes to be analysed.

### 2.6. Data Management

Data will be analysed within a secure online environment (VALT) facilitated by the Centre for Victorian Data Linkage. Datasets to be linked within the environment include the GenV and Me data, the Australian Immunisation Registry (AIR), the Victorian Emergency Minimum Dataset (VEMD), and the Victorian Admitted Episodes Dataset (VAED). Access is limited to authorised study personnel, and all data will be de-identified prior to analysis. Only results generated from deidentified data will be extracted. Data management procedures will comply with institutional governance requirements and applicable data protection standards.

### 2.7. Ethics

Human Research Ethics Committee approval has been granted for both GenV (RCH HREC 2019.011) and the Maternal Vaccine study outlined in this protocol (RCH HREC 2023.91383).

## 3. Conclusions

Further knowledge around the developmental, health, and allergy outcomes for infants after maternal vaccination during pregnancy with COVID-19 and influenza vaccines is needed to answer questions about long-term vaccine safety for parents and providers. These longer-term outcomes have not previously been described. The results of this study will offer value to clinicians through the provision of comprehensive data on health, developmental, and allergy outcomes of infants up to 12 months of age. The reporting of developmental data will be a new contribution to knowledge around outcomes after vaccination during pregnancy.

## Figures and Tables

**Table 1:** Demographic mother–baby descriptor tables.

Demographic	Statistic	Comments
Number of mother–baby pairs. Divide by exposure groups	n (%)	
Sex of baby	n (%)	
Parental country of birth	n (%)	P1, P2
Ethnicity	n (%)	P1, P2
Highest level of education attained by each guardian	n (%)	P1, P2
Aboriginal or Torres Strait Islander status	n (%)	P1, P2
Parental global health before and during pregnancy (CHQ-PF50)	n (%)	P1, P2
Household composition		P1
Reside with child at 6 months of age		P1, P2

P1: primary guardian; P2: other guardian.

**Table 2:** Exposure and comparator groups.

Exposure and Comparator Groups	Mother–Baby Pairing Group Description
Vaccinated cohort 1	Mother receives a COVID-19 vaccine during pregnancy. Includes women who may or may not have received a pertussis vaccine during pregnancy.
Vaccinated cohort 2	Mother receives an influenza vaccine during pregnancy. Includes women who may or may not have received a pertussis vaccine during pregnancy.
Vaccinated cohort 3	Mother receives a COVID-19 and an influenza vaccine during pregnancy. Includes women who may or may not have received a pertussis vaccine during pregnancy.
Unvaccinated cohort	Pregnant women who do not receive any vaccine during pregnancy
Vaccinated cohort, non-respiratory virus	Pregnant women who only receive a pertussis containing vaccine during pregnancy

**Table 3:** Core study outcomes to be investigated.

Domain	Outcomes to Be Investigated	Data Time Point (Months)	Dataset
Perinatal and infant health	Preterm birth: birth prior to 37 weeks of gestation	3 9	VAED GenV dataset
	Major congenital malformation: malformation identified by parents and/or health practitioners within the capabilities of the GenV dataset	3, 6, 9, 12	VAED ICD-10-AM
	Small for gestational age: infant born at <10th centile for gestational age	3	VAED ICD-10-AM code P05.1
	Neonatal hospitalisation within the first 28 days of life	1	VAED, VEMD
Childhood health	Food allergy: type of food, symptoms, timing, avoidance, diagnosis, diagnostic testing	3, 6, 9, 12	GenV dataset
	Drug allergy	3, 6, 9, 12	GenV dataset
	Atopy; eczema: skin rash, itch, onset, duration, severity, treatment; nappy rash; wheeze	3, 6, 9, 12	GenV dataset
	Allergy resulting in a presentation to a Victorian Hospital	1–12	VAED and VEMD ICD-10-AM: T78.0, T78.1, T78.2, T78.4, L27.2, L23.6, K52.2, Y37.0 *, Z88 *, Z91 *
	Medical conditions: defined as any medical condition identified by parents or healthcare providers within the GenV dataset at data collection time-points 3, 6, 9, and 12 months Domains include:	3, 6, 9, 12	Genv dataset
	Neurological Febrile seizures Epilepsy or other seizure condition	3, 6, 9, 12	GenV dataset VAED and VEMD ICD-10-AM: R56.0 *, G40 *, A80-89
	Hospitalisation during the first 12 months of life	1–12	ICD-10-AM VAED
Infant development Gross motor	Tummy time Head control unsupported Push to elbows, roll front to back, roll in both directions, sit unsupported Baby feeds themselves Stand whilst holding a support Crawling: hands and knees; hands and feet, legs straight; belly crawling (pulling forward on tummy) Sitting and “bottom scooting” Rolling Never crawled, went straight to standing and pushing Walking independently	3, 6 3 6 9 12	GenV dataset
Fine motor	Fix and follow with eyes Pincer grip	3 9	GenV dataset
Social skills	First started to smile, first stated to laugh Shy with other people Look for hidden objects Plays “peek a boo” or “pat a cake” Follow one step directions Pretend play	3 9 9 12	GenV dataset
Speech	Single sounds “ma”, “ga” Two syllable sounds Name calling: mamma, dadda Uses words or sounds to get attention (3 point scale)	6 9 12 12	GenV dataset
Hearing	Hearing loss, ear infection, other	3, 6, 9, 12	GenV dataset
Vision	Low vision, squint or strabismus, eye infection, other	3, 6, 9, 12	GenV dataset
Growth	Weight Head circumference Length	0, 6, 12 0, 12 0, 6, 12	GenV dataset

AIR: Australian Immunisation Register; VAED: Victorian Admitted Episode Dataset; VEMD: Victorian Emergency Minimum Dataset. * This is a way the coding system encompasses all codes that follow Y37.0 (e.g., Y37.01, Y37.02) etc.

**Table 4:** Approach to missing data.

Data Items	Risk Assessment and Approach to Missing Data
Maternal receipt of a COVID-19, influenza, or pertussis vaccination status from the AIR	Minimal risk due to data linkage processes If maternal vaccination status is unable to be ascertained by AIR, the participant will be excluded.
Maternal and infant demographics	Minimal risk: data collected during recruitment survey
Infant gestational age at the time of birth using ICD-10-AM [19] administrative coding from VAED [18]	Minimal due to data linkage processes If ascertainment of gestational age at birth is unavailable via VAED, the gestational age entered by parents in the GenV and Me survey at 9 months will be used. If the GenV and Me survey data entry does not include gestational age, and the participant is unable to be linked to the VAED, they will be excluded
Developmental outcomes collected at 3, 6, 9, and 12 months of age via GenV and Me [16]	Moderate risk. Relies on guardian input at 4 separate time points. To be included for the assessment of the primary outcome, questions relating to development must be input by a guardian at 12 months. If a participant has missing data at some but not all time points, they will be included in a sensitivity analysis, with their final data entry point for developmental assessment being the time point developmental analysis will be conducted to
Health and allergy outcomes collected at 3, 6, 9, and 12 months of age via GenV and Me [16]	Moderate risk. Relies on guardian input at 4 separate time points.
Health and allergy outcomes. Defined by administrative coding endpoints (ICD-10-AM) [19] within VAED [18] and VEMD [20]	Minimal risk. Outcomes ascertained through data linkage

AIR: Australian Immunisation Register; VAED: Victorian Admitted Episodes Dataset; VEMD: Victorian Minimum Emergency Dataset.

## Data Availability

Data will be available for review in the published results paper.

## Supplementary Materials

The following supporting information can be downloaded at: https://www.mdpi.com/article/10.3390/vaccines14050449/s1, File S1: ICD-10-AM Preterm birth Codes to be extracted from the Victorian Admitted Episodes Dataset.
