# Supplementary material for: The Maternal Vaccine Study Protocol: A Victorian Cohort Study Evaluating Infant and Childhood Safety and Health and Developmental Outcomes After Vaccination Against Respiratory Viruses in Pregnancy
Source: Vaccines (Basel). 2026 May 18;14(5):449. doi: 10.3390/vaccines14050449 (PMC13211398; doi:10.3390/vaccines14050449)
Supplement: Supplementary file 1 [file vaccines-14-00449-s001.zip › vaccines-4316197-supplementary.pdf]

Supplementary Materials

ICD-10-AM Preterm birth Codes to be extracted from the Victorian Admitted Episodes Dataset

Version 11 (from study onset until 30th June, 2022)

ICD-10-AM code	Code definition
P0721	Extreme immaturity of newborn, gestational age less than 24 completed weeks
P0722	Extreme immaturity of newborn, 24 or more completed weeks, but less than 28
P0730	Preterm infant, unspecified
P0731	Preterm infant, 28 or more completed weeks but less than 32 completed weeks
P0732	Preterm infant, 32 or more completed weeks, but less than 37 completed weeks

Version 12 (commencing 1st July, 2022)

ICD-10-AM Code	Code definition
P0740	Extreme preterm birth, unspecified gestational age
P0741	Extreme preterm birth, gestational age less than 22 completed weeks
P0742	Extreme preterm birth, gestational age 22 completed weeks
P0743	Extreme preterm birth, gestational age 23 completed weeks
P0744	Extreme preterm birth, gestational age 24 completed weeks
P0745	Extreme preterm birth, gestational age 25 completed weeks
P0746	Extreme preterm birth, gestational age 26 completed weeks
P0747	Extreme preterm birth, gestational age 27 completed weeks
P0750	Preterm infant, unspecified
P0751	Preterm infant, gestational age 28 completed weeks
P0752	Preterm infant, gestational age 29 completed weeks
P0753	Preterm infant, gestational age 30 completed weeks
P0754	Preterm infant, gestational age 31 completed weeks
P0755	Preterm infant, gestational age 32 completed weeks
P0756	Preterm infant, gestational age 33 completed weeks
P0757	Preterm infant, gestational age 34 completed weeks

P0758	Preterm infant, gestational age 35 completed weeks
P0759	Preterm infant, gestational age 36 completed weeks
